# Effects and efficacy of laparoscopic fundoplication in children with GERD: a prospective, multicenter study

**DOI:** 10.1007/s00464-016-5070-z

**Published:** 2016-07-01

**Authors:** Femke A. Mauritz, J. M. Conchillo, L. W. E. van Heurn, P. D. Siersema, C. E. J. Sloots, R. H. J. Houwen, D. C. van der Zee, M. Y. A. van Herwaarden-Lindeboom

**Affiliations:** 1 Department of Pediatric Surgery, Wilhelmina Children’s Hospital, University Medical Center Utrecht, Room: KE.04.140.5, PO Box 85090, 3508 AB Utrecht, The Netherlands; 2 Department of Pediatric Gastroenterology, Wilhelmina Children’s Hospital, University Medical Center Utrecht, Utrecht, The Netherlands; 3 Department of Gastroenterology and Hepatology, University Medical Center Utrecht, Utrecht, The Netherlands; 4 Department of Gastroenterology and Hepatology, Maastricht University Medical Center, Maastricht, The Netherlands; 5 Department of Pediatric Surgery, Emma Children’s Hospital, Amsterdam Medical Center, Amsterdam, The Netherlands; 6 Department of Pediatric Surgery, Erasmus MC - Sophia Children’s Hospital, Rotterdam, The Netherlands

**Keywords:** Pediatric, Children, Reflux, GERD, Fundoplication, Efficacy

## Abstract

**Introduction:**

Laparoscopic antireflux surgery (LARS) in children primarily aims to decrease reflux events and reduce reflux symptoms in children with therapy-resistant gastroesophageal reflux disease (GERD). The aim was to objectively assess the effect and efficacy of LARS in pediatric GERD patients and to identify parameters associated with failure of LARS.

**Methods:**

Twenty-five children with GERD [12 males, median age 6 (2–18) years] were included prospectively. Reflux-specific questionnaires, stationary manometry, 24-h multichannel intraluminal impedance pH monitoring (MII-pH monitoring) and a ^13^C-labeled Na-octanoate breath test were used for clinical assessment before and 3 months after LARS.

**Results:**

After LARS, three of 25 patients had persisting/recurrent reflux symptoms (one also had persistent pathological acid exposure on MII-pH monitoring). New-onset dysphagia was present in three patients after LARS. Total acid exposure time (AET) (8.5–0.8 %; *p* < 0.0001) and total number of reflux episodes (*p* < 0.001) significantly decreased and lower esophageal sphincter (LES) resting pressure significantly increased (10–24 mmHg, *p* < 0.0001) after LARS. LES relaxation, peristaltic contractions and gastric emptying time did not change. The total number of reflux episodes on MII-pH monitoring before LARS was a significant predictor for the effect of the procedure on reflux reduction (*p* < 0.0001).

**Conclusions:**

In children with therapy-resistant GERD, LARS significantly reduces reflux symptoms, total acid exposure time (AET) and number of acidic as well as weakly acidic reflux episodes. LES resting pressure increases after LARS, but esophageal function and gastric emptying are not affected. LARS showed better reflux reduction in children with a higher number of reflux episodes on preoperative MII-pH monitoring.

Gastroesophageal reflux disease (GERD) frequently occurs in the pediatric population [1, 2]. In severe GERD resistant to medical treatment, laparoscopic antireflux surgery (LARS) can be warranted [2–4]. Many studies have been published on pediatric LARS [5–12]. Most of these studies had a retrospective design and could only conclude that the procedure resulted in symptom control in 57–100 % of patients [11, 13, 14]. To assess the efficacy of LARS, it is important to use validated questionnaires before and after LARS. Such questionnaires, however, have not been used in the majority of pediatric LARS studies [15]. In addition to evaluation of reflux symptoms, more objective assessments of (acid) reflux, such as multichannel intraluminal impedance pH (MII-pH) monitoring, should be performed [9, 10, 16].

In previous published pediatric studies, objective assessments were primarily performed using only pH monitoring [6, 9, 10, 12]. MII-pH monitoring enables quantification of both acid and weakly acidic reflux and the proximal extent of the refluxate [17] and therefore increases the yield of symptom association assessments in children [18]. Studies in children using MII in addition to pH monitoring so far either were retrospective [19] or only investigated efficacy in a selected patient population [20]. However, up to now none of the studies quantifying both reflux symptoms and more objective assessments of GERD after LARS have shown a correlation between both measurements [9, 10]. It is therefore important to evaluate effects and efficacy of LARS using both validated questionnaires and objective assessment tools.

The success of LARS is determined by the disappearance or reduction of GERD, but is also influenced by postoperative sequelae, such as severe dysphagia and gas/bloating [21]. It is therefore indicated to identify predictors for failure in order to enable optimal preoperative counseling on the risk of persisting or recurrent GERD after LARS. Rosen et al. [19] addressed this issue in a retrospective study by trying to identify predictors for failure of LARS in children using preoperative MII-pH monitoring in 37 patients. However, no predictors could be identified in this study.

The aim of the current prospective study was to objectively assess the effect and efficacy of LARS in pediatric patients and to identify predictors of LARS failure.

## Methods

We performed a prospective multicenter study in three University Medical Centers in the Netherlands that are specialized in performing laparoscopic fundoplication in children (Wilhelmina Children’s Hospital, University Medical Center Utrecht (UMCU); Sophia’s Children’s Hospital, Erasmus University Medical Center (Erasmus MC) and Maastricht University Medical Center (MUMC). From July 2011 to December 2013 we prospectively included all pediatric patients diagnosed with PPI therapy-resistant GERD. Patients who underwent any previous esophageal or gastric surgery (except previous gastrostomy placement) and those with structural abnormalities other than an esophageal hiatal hernia were excluded.

### Surgical procedures

All laparoscopic fundoplication procedures were performed by experienced pediatric surgeons. In the UMCU Utrecht, the anterior, partial fundoplication according to Thal [22] was used to perform fundoplication. In the other two UMCs (EMC and MUMC), the posterior, total fundoplication according to Nissen [23] was performed. Before fundoplication, the distal esophagus was fully mobilized; the distal 3 cm of the esophagus was repositioned back into the abdomen. Both vagal nerves were identified, and a crusplasty was performed routinely (UMCU and EMC). Thereafter, the fundoplication was constructed. The Thal fundoplication was performed by plicating the fundus of the stomach over 270° against the distal anterior intra-abdominal part of the esophagus and the diaphragmatic crus [9, 22]. A floppy Nissen was constructed with one of the sutures of the 360 degrees posterior wrap incorporated in the esophageal wall [23].

### Clinical assessment

Before and 3 months after laparoscopic fundoplication, clinical assessment was performed using stationary manometry, 24-h multichannel intraluminal impedance pH monitoring (MII-pH monitoring), ^13^C-labeled Na-octanoate breath test and a reflux-specific symptom questionnaire. Surgical reinterventions, type and indication for reintervention, endoscopic procedures, use of antireflux medication, complications and comorbidities were registered in a prospective database.

#### Reflux-specific symptom questionnaires

To assess reflux symptoms, patients and/or their parents were asked to fill out the validated age-adjusted Gastroesophageal Reflux Symptom Questionnaire (GSQ) before and after LARS [24]. Reflux symptoms and dysphagia were scored for frequency and severity on a score ranging from 1 (none) to 7 (most severe). Symptoms were defined as no symptoms (no symptoms reported); mild (mild symptoms weekly); moderate (mild symptoms daily or severe symptoms weekly) and severe (severe symptoms daily). Reflux symptoms were scored using the symptoms heartburn, regurgitation, food refusal and vomiting. Furthermore, the need for acid suppressive therapy after LARS was registered.

#### Nutritional status

Weight and height measurements were converted to weight-for-length and length-for-age *z* scores based on the Netherlands Organization for Applied Scientific Research (TNO) growth standards [25]. *Z* scores allow comparison of an individual’s weight or height, adjusting for age and sex relative to a reference population, expressed in standard deviations from the reference mean.

#### Stationary manometry

For esophageal stationary manometry, age-adjusted stationary water-perfused sleeve-manometry catheters were used (Mui Scientific, Mississauga, Ontario, Canada). The sleeve-manometry catheter was positioned with the sleeve at the level of the lower esophageal sphincter (LES) using the pull-through technique. In a semi-recumbent position, patients received 10 liquid bolus challenges using saline combined with lemonade (5 ml) in order to study the manometric response. During the study, data were recorded on the Stationary Solar Gastro System (Medical Measurement Systems, Enschede, The Netherlands). Manometry tracings were analyzed for LES resting and nadir pressure, LES relaxations, number of peristaltic contractions and peak amplitude of the contractions according to previously accepted standards [26].

#### Ambulatory 24-h MII-pH monitoring

Ambulatory 24-h MII-pH testing was conducted after cessation of all medication that may have an effect on gastrointestinal motility and acid secretion for at least 3 days. MII-pH monitoring was performed using an age-adjusted combined impedance pH catheter with six impedance segments and one ISFET pH electrode (Unisensor AG, Attikon, Switzerland). The pH electrode was positioned above the upper border of the manometrically localized lower esophageal sphincter. Impedance and pH signals were stored on a digital data logger (Ohmega, Medical Measurement Systems, Enschede, The Netherlands), using a sampling frequency of 50 Hz. Patients were instructed to record reflux symptoms, supine resting periods and meals, including drinks, in a diary and by marking the symptom using the recording button on the data logger. The 24-h MII-pH tracings were analyzed for the number and acidity of reflux episodes according to previously described definitions [17]. Pathological acid exposure was defined as total acid exposure time (AET) ≥6 %, ≥9 % in upright and ≥3 % in the supine body position [27, 28]. The symptom index (SI) and the symptom association probability (SAP) were calculated if patients had experienced symptoms during the measurement [29, 30].

#### Gastric emptying breath test

To assess gastric emptying (GE) half-time, we used a ^13^C-labeled Na-octanoate breath test [31]. Subjects fasted for at least 6 h before the study. In children >4 years of age, a solid test meal containing ^13^C-labeled Na-octanoate was performed with 375-g pancake containing 45 mg of ^13^C-labeled Na-octanoate (a stable isotope).

For younger children or children who were unable to eat the pancake within 15 min, 100 mg of ^13^C-labeled Na-octanoate was added to a liquid formula (infant formula, full cream milk or chocolate milk). Breath samples were obtained in duplicate at 15-min intervals during the course of 4 h (for the liquid test, breath samples were obtained at 5-min intervals during the first 30 min). Hereafter, the ratio between ^12^CO_2_ and ^13^CO_2_ content in breath samples was analyzed with an isotope ratio mass spectrometer. Finally, three parameters were calculated. Gastric half-emptying time (GGE-T½) was defined as the time when the first half of the ^13^C-labeled substrate had been metabolized, that is, when the cumulative excretion of ^13^C in the breath was half the ingested amount. Gastric emptying percentiles (*P*) were calculated according to the reference values obtained by van den Driessche et al. [32]. GE percentiles higher than 75 were considered delayed and above 95 severely delayed. The gastric emptying coefficient (GEC) reflects a global index for GE, influenced by both the rate of appearance and disappearance of ^13^C in breath.

### Sample size calculation

A sample size of 50 patients was calculated based on the assumption that approximately 20 % of pediatric GERD patients will fail after LARS. Success of LARS was defined as: (1) complete symptom relief and normalized MII-pH monitoring or (2) complete symptom relief and near-normal MII-pH monitoring or (3) normalized MII-pH monitoring combined with a significant improvement of reflux symptoms (complaints less than moderate/weekly). Using the logistic regression model according to Frank Harrell [33], five failures were required to reliably identify a determinant of failure. Determinant of interest was gastric emptying and age at time of operation.

### Patients

In total 25 children were included in our study. After enrollment of the 25th patient, the study was stopped prematurely, because the inclusion rate was lower than anticipated. Mean age of the included patients was 6 (range 2–18) years at the time of fundoplication (Table 1). Five children (80 %) had normal neurodevelopment (NN), while impaired neurodevelopment (NI) was seen in five children (20 %). Cause of NI is shown in Table 2.

**Table 1:** Baseline characteristics

	(Median; IQR)
Age at time of operation (years)	6.0 (3.0–11.0)
Duration of hospital admission (days)	3.0 (2.0–4.5)

	*n* (%)
Male gender	12 (48.0 %)
Impaired neurodevelopment	5 (20.0 %)
Gastrostomy preoperatively in situ	4 (16.0 %)

**Table 2:** Impaired neurodevelopment (*n* = 5)

CHARGE syndrome
Mitochondrial complex II deficiency
Posthypoxic encephalopathy
Congenital rubella infection
Impaired neurodevelopment of unknown origin with autistic behavior

### Ethical approval and trial registration

This study was registered at the start of the study in the Dutch national trial registry (www.trialregister.nl; Identifier: 2934). Ethical approval for this prospective multicenter study was obtained from the University Medical Center Utrecht Ethics Committee, and local approval was obtained by the remaining two participating centers. Prior to study procedures, informed consent from the patients’ parents and children (≥12 years) was obtained.

### Statistical analysis

Continuous variables, when symmetric, were expressed as mean ± standard error. Skewed variables were expressed as median with interquartile ranges (IQR). For statistical analysis, we used the paired sample *t* test or the Wilcoxon signed-rank test, whenever appropriate. The McNemar–Bowker test was used to compare groups in case of nominal outcome measures. Exploratory subgroup analysis for all outcome measures was performed comparing neurodevelopment and type of fundoplication. The primary aim was to perform a logistic regression analysis if sufficient LARS failures were identified. Linear regression analysis was performed to identify determinants influencing the effect of LARS on reflux control measured by 24-h MII-pH monitoring. Determinant of interest was age at time of operation, neurodevelopment, type of fundoplication, preoperative number of reflux episodes on 24-h MII-pH monitoring and preoperative gastric emptying rate. Differences with a *p* < 0.05 were considered statistically significant. All analyses were performed using IBM^®^22.0.0 SPSS statistical package (IBM, Armonk, NY).

## Results

**Fig. 1:**
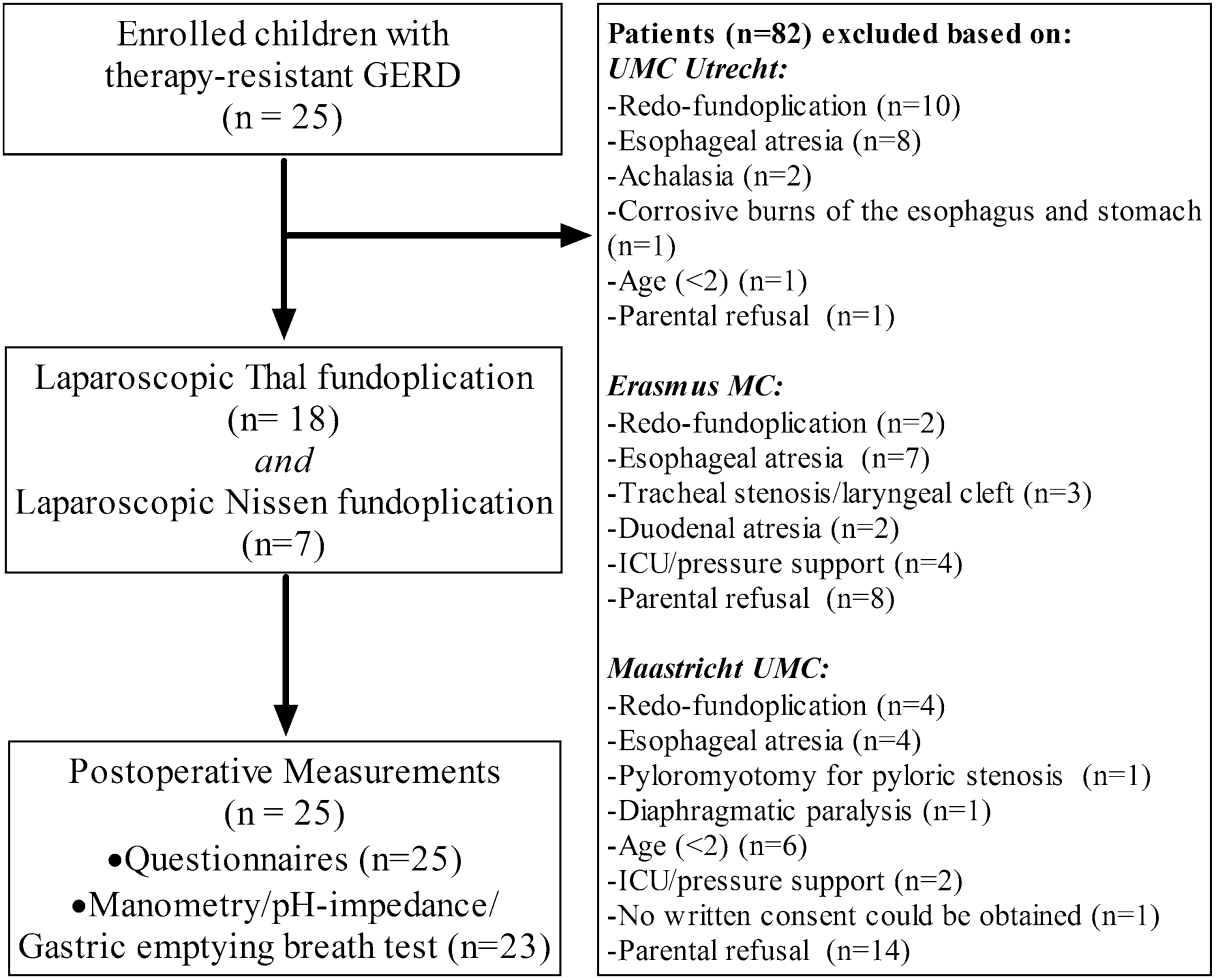
Flowchart of patient selection and enrollment

In total 18 Thal and 7 Nissen fundoplications were performed (Fig. 1). In all patients, fundoplication could be completed by laparoscopy. Perioperative complications were not observed. Median hospital admission time was 3.0 (2.0–4.5) days (Table 1). In one patient with retching based on impaired neurodevelopment a redo-fundoplication was indicated because of severe recurrent reflux (pathological reflux on 24-h pH monitoring and severe reflux symptoms) caused by hiatal herniation. Another patient required emergency gastroscopy to remove a food bolus impacted in the esophagus 1 day after LARS. In six children temporary nasogastric tube feeding was required to obtain sufficient caloric intake. Insufficient caloric intake was caused by transient dysphagia (*n* = 4), persistent dysphagia (*n* = 1) or rejection of oral feedings without dysphagia (*n* = 1).

### Symptom assessment

All patients and/or parents completed the reflux-specific symptom questionnaire (Fig. 1). Overall reflux symptoms significantly decreased after LARS (*p* = 0.001). In three of 25 (12 %) patients, reflux symptoms persisted (1/3 also had persistent pathological acid exposure on MII-pH monitoring) (Table 3). The use of acid suppressive medication decreased from 100 % of all patients preoperatively to 16 % (*n* = 4) after operation. Analysis in subgroups comparing children with NI to NN [NN (5 %) vs NI (40 %); *p* = 0.099] and Nissen compared to Thal fundoplication [Nissen (11 %) vs Thal (17 %); *p* = 0.597] did not show significant differences in the presence of GERD symptoms after LARS.

**Table 3:** Symptom assessment

	Preoperative (*n*, %)	3–4 months postoperative (*n*, %)	*p* value
Reflux symptoms			
None	0 (0 %)	17 (68 %)	0.001
Mild	2 (8 %)	5 (20 %)	
Moderate	7 (28 %)	2 (8 %)	
Severe	16 (64 %)	1 (4 %)	
Dysphagia			
None	13 (52 %)	15 (60 %)	0.887
Mild	4 (16 %)	3 (12 %)	
Moderate	3 (12 %)	3 (12 %)	
Severe	5 (20 %)	4 (16 %)	

*n* number of patients, *%* percentage of patients, *p* < 0.05 is considered significant

Moderate-to-severe dysphagia was reported in eight (32 %) patients before LARS and in seven (28 %) patients 3 months after LARS (*p* = 0.887) (Table 3). New-onset dysphagia was seen in three of these seven patients with dysphagia after LARS. Dysphagia more frequently occurred in NI children [NI (80 %) vs NN (15 %); *p* = 0.012] after LARS compared to NN patients. Furthermore, there was a trend showing that children undergoing Nissen fundoplication had more frequently dysphagia compared to those undergoing Thal fundoplication [Nissen (57 %) vs Thal (17 %); *p* = 0.066].

### Nutritional status

Height-for-weight [−0.2 SD (−1.0 to 0.7) to −0.5 SD (−1.3 to 0.1); *p* = 0.57] and height-for-age [−0.9 SD (−1.2 to 0.1) to −1.0 SD (−1.5 to 0.4); *p* = 0.42] scores remained similar when comparing preoperative to 3-month postoperative measurements.

### Clinical assessment tests

Postoperative manometry, 24-h MII-pH monitoring and gastric emptying breath test were not performed in two patients because of parental refusal (Fig. 1). LES resting pressure significantly increased after fundoplication from 10 mmHg (7–18) to 24 mmHg (17–26), *p* < 0.0001. Nadir LES pressure also significantly increased from 0 mmHg (0–8) to 3.5 mmHg (0–8) after LARS, *p* < 0.0001. Complete LES relaxation, percentage of continued peristaltic contractions and peak amplitude all remained similar (Table 4). Subgroup analysis showed no differences comparing NI to NN children. Children undergoing Thal fundoplication had a significantly higher preoperative LES resting pressure compared to those who underwent Nissen fundoplication [Thal (14.6 mmHg) vs Nissen (6.5 mmHg); *p* = 0.001]; however, after LARS no statistical difference was found [Thal (22.7 mmHg) vs Nissen (19.8 mmHg); *p* = 0.525]. All other manometry outcome measures were similar when comparing Thal to Nissen fundoplication.

**Table 4:** Results of clinical assessment tests

	Preoperative (IQR)	3–4 months postoperative (IQR)	*p* value
24-h MII-pH measurement			
Total GER episodes	91.5 (8–230)	14 (2–153)	<0.0001
Acid GER episodes	61.5 (34.3–93.8)	8 (1–13)	<0.0001
Weakly acid GER episodes	23 (10.5–42)	5 (3–11)	0.002
Liquid GER episodes	55.5 (11–153)	10 (2–96)	<0.0001
Mixed GER episodes	37.5 (3–176)	3 (0–57)	<0.0001
Proximal extend			
Z1 (proximal esophagus)	26.5 (14.5–55.3)	2 (0–8)	<0.001
Z3 (mid esophagus)	75.5 (64.8–88)	57 (44–71)	0.009
Z5 (distal esophagus)	100 (100–100)	100 (100–100)	NA
Total acid exposure (%)	8.5 (2.5–32.8)	0.8 (0–2.8)	<0.0001
Longest reflux episode (min)	20.7 (3.4–66.7)	3.8 (0–21.6)	<0.0001
SI (%)	75 (18.8–100)	50 (0–100)	0.111
SAP (%)	100 (97.3–100)	93.2 (22.1–98.7)	0.048
Stationary manometry			
LES resting pressure (mmHg)	10 (6.5–18)	23.5 (17–26)	<0.0001
LES nadir pressure (mmHg)	0 (0–8)	3.5 (0–8)	<0.0001
Complete LES relaxation (%)	100 (100–100)	100 (100–100)	0.311
Continued peristaltic contraction (%)	100 (100–100)	100 (100–100)	0.149
Peak amplitude (mmHg)	74 (39–109)	66 (24–139)	0.299
Gastric emptying test			
Gastric emptying half-time (min)	76.5 (49.3–89)	56 (47–78)	0.102
Gastric emptying percentile	75 (0–99)	70 (2–99)	0.530
GEC	3.0 (2.5–5.6)	3.6 (2.3–4.7)	0.463

*GER* gastroesophageal reflux, *SI* symptom index, *SAP* symptom association probability, *NA* not applicable, *LES* lower esophageal sphincter, *GEC* gastric emptying coefficient, *IQR* interquartile range, *p* < 0.05 is considerd significant

Twenty-four hour MII-pH monitoring showed a significant decrease in total acid exposure time and number of reflux episodes (*p* < 0.001; Table 4). Acidic, weakly acidic, liquid and mixed reflux episodes also decreased significantly (Table 4). In two patients pathological reflux persisted after LARS, although in one of these patients total acid exposure time (AET) decreased from 32.8 % (severe pathological) to 9.7 % (near-normal). Subgroup analysis comparing NI to NN children revealed that preoperative acid exposure time and total number of reflux episodes (RE) before LARS were significantly higher in NI children [AET: NN (9.5 %) vs NI (19.3 %); *p* = 0.006 and RE: NN (91.5) vs NI (181.4); *p* = 0.002]. After LARS NI children still had more reflux, although it was not statistically significant [AET: NN (1.2 %) vs NI (7.2 %); *p* = 0.22 and RE: NN (16.6) vs NI (41.8); *p* = 0.42]. Other 24-h MII-pH outcome measures were similar comparing NI to NN children. Comparing Thal to Nissen fundoplication only identified a significant difference in preoperative total number of reflux episodes on 24-h MII-pH monitoring [AET: Thal (121.4) vs Nissen (76.7); *p* = 0.03]; however, after LARS outcomes were not significantly different [AET: Thal (25.9) vs Nissen (11.4); *p* = 0.33).

**Fig. 2:**
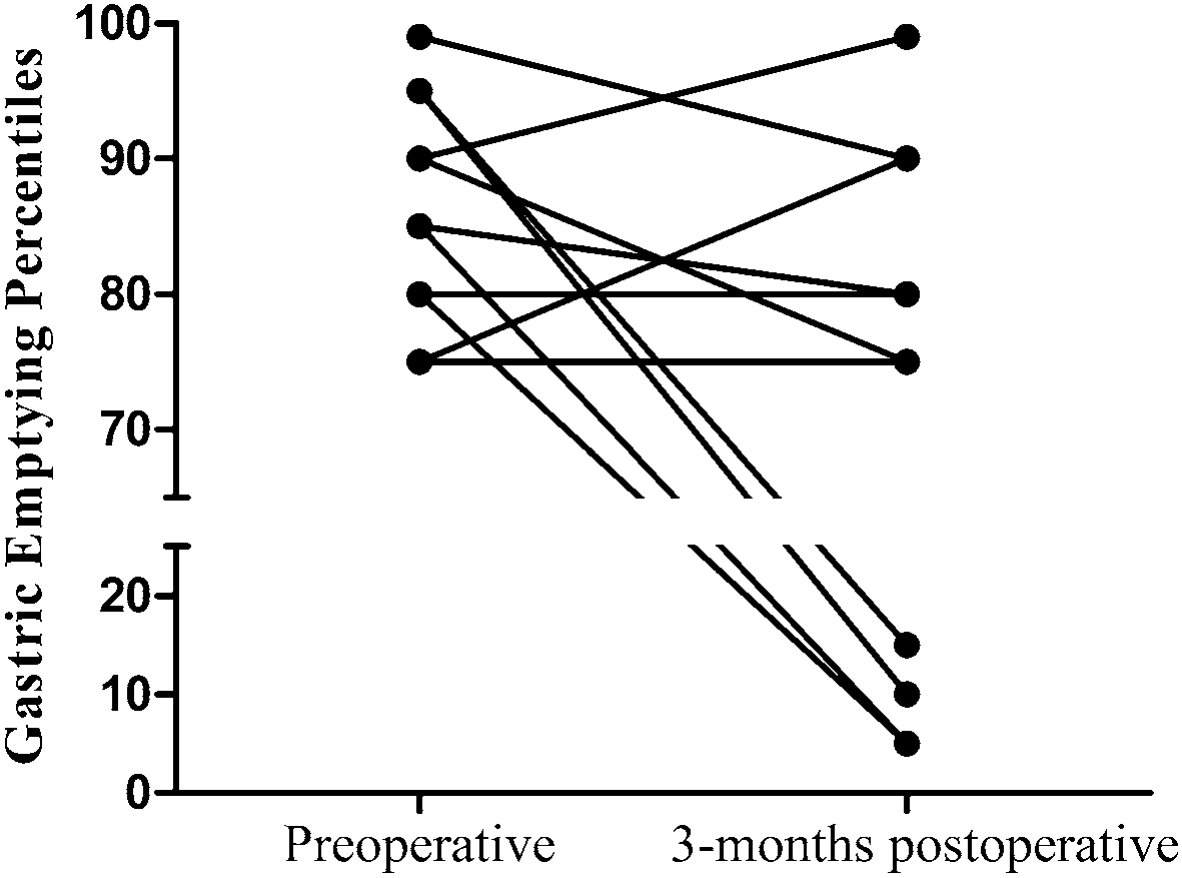
Effect of LARS on gastric emptying in patients with preoperative delayed gastric emptying

GE half-time [77 min (0–113) to 56 min (14–103); *p* = 0.102] and GE percentiles did not significantly change after LARS (Table 4). However, looking at a subset of patients with preoperative delayed (*n* = 13) or severely delayed (*n* = 8) GE, GE half-time [84 min (58–106) to 54.4 min (40.3–87.3); *p* = 0.023] and GE percentiles [85 (75–95) to 75 (10–85); *p* = 0.029] improved significantly. Furthermore, in four patients GE normalized after LARS (Fig. 2). Subgroup analysis comparing gastric emptying in NI to NN children and Thal to Nissen fundoplication did not show any significant differences.

### Predictors of LARS failure

After LARS only one patient had persistent reflux symptoms and pathological reflux. As only one patient failed LARS, a logistic regression analysis was therefore not feasible.

### Predictors of the effect of LARS on reflux control

Linear regression analysis identified preoperative reflux episodes on MII-pH monitoring as a determinant influencing the effect of LARS reflux episodes (estimate = 0.791; *p* < 0.0001). Age at the time of operation, neurodevelopment, type of fundoplication and GE did not show any significant effect (Table 5).

**Table 5:** Predictors of the effect of LARS on reflux reduction

	Estimate	*p* value	95 % CI
Age at time of operation	−6.1	0.76	−47.2 to 34.9
Neurodevelopment	0.8	0.61	−2.7 to 4.4
Type of fundoplication	3.4	0.85	−33.8 to 40.6
Preoperative total number of reflux episodes	0.8	<0.0001	0.5 to 1.1
Preoperative gastric emptying	−0.2	0.34	−0.6 to 0.2

Linear regression analysis (*95* *% CI* 95 % confidence interval)

## Discussion

In the present study, LARS was successful in 96 % of children with therapy-resistant GERD according to both the symptom and clinical response. LARS reduced not only acidic reflux episodes, but weakly acidic reflux was also significantly reduced.

Reflux symptoms significantly decreased after LARS, and in three (12 %) patients reflux symptoms persisted at 3-month follow-up. This short-term success rate is similar to other prospective studies in pediatric antireflux surgery [7, 11, 12]. Subgroup analysis showed that the incidence of persistent or recurrent reflux symptoms was similar after both Thal and Nissen fundoplication (*p* = 0.597). A recent meta-analysis in both adults and children also reported no differences between partial and complete fundoplication techniques in reduction of reflux symptoms [34, 35]. It must be noted that our study was not powered to study the differences between both techniques and therefore results may differ in a larger study population.

When comparing reflux symptoms after LARS in children with NI to children with NN, we found that reflux symptoms were present in only 5 % (1/20) of NN patients versus almost half (40 %; 2/5) of NI children. This difference was not statistically different possibly because the current study was not powered to identify differences between both groups. Before LARS total acid exposure time and number of reflux episodes were significantly higher in NI children; however, after LARS no significant differences were observed. Some authors hypothesized that NI children may insufficiently benefit from LARS [5, 36, 37]; however, we found no statistical significant differences in our study.

Only one of the three patients with persistent reflux symptoms also had pathological acid exposure on MII-pH monitoring; conversely, only one of the two (both NI) patients with pathological reflux had reflux symptoms after LARS. In the other NI patient, reflux symptoms completely resolved and 24-h MII-pH monitoring decreased to near-normal acid exposure. In adults a lack of correlation between reflux symptoms and objective assessment of the prevalence of (acid) refluxate in the esophagus has been reported as well [38, 39]. It is thought that recurrent or persistent symptoms may be caused by concomitant functional disease such as functional dyspepsia or hypersensitivity [39]. Moreover, in NI patients symptom assessment may be even more challenging because NI children are frequently verbally restricted and often have more (co-)morbidity, which underscores the importance of objective assessment of GERD in these children.

Objective assessment of reflux using 24-h MII-pH monitoring showed that LARS resulted in a significant decrease in acidic and also weakly acidic reflux. An earlier published pilot study by Loots et al. [8] did not show significant reduction in weakly acidic reflux. However, in this study only 10 patients were included, which may result in a type II error. Weakly acidic reflux is often not successfully treated by acid suppressive therapy (i.e., proton pump inhibitors) as it only decreases the acidity of the refluxate but does not treat the actual retrograde movement of gastric content [40]. Furthermore, in young children gastric content is buffered by frequent feeds and is therefore often not acidic.

LES resting and nadir pressure increased significantly after LARS, which is in accordance with previous studies on pediatric antireflux surgery [12, 36]. Increase in the esophagogastric junction competence is expected, as it is one of the mechanisms in which LARS prevents GERD [41–43]. It has been reported that LARS may affect LES relaxations and esophageal motility, thereby inducing postoperative dysphagia [44]. In this current study, LARS did, however, not affect LES relaxations and esophageal motility.

In seven patients dysphagia was found after LARS. New-onset dysphagia was seen in three of these patients and was significantly more prevalent in NI children. Furthermore, a nonsignificant trend was shown in favor of Thal fundoplication (17 vs 57 %), compared to Nissen fundoplication. New-onset dysphagia is thought to be caused by fundoplication-induced restriction and postoperative swelling at the esophagogastric junction. LES pressure testing in our cohort did show a significant increase in LES resting and nadir pressure, which may reflect this restriction. Complete (e.g., Nissen) and partial (e.g., Thal and Toupet) fundoplications are all currently used in the pediatric population, and reported dysphagia rates differ between these techniques, but are mostly less prominent after partial fundoplication [7, 15, 35]. Finally, dysphagia may be a manifestation of GERD, as dysmotility of the distal esophagus is frequently seen in adult patients with esophagitis [45, 46].

In the current study, only one patient failed after LARS, which made a logistic regression analysis for the identification of predictors of LARS failure not possible. Rosen et al. also used MII-pH monitoring trying to identify predictors for LARS failure using a Cox regression analysis; however, their study was underpowered with only 37 patients and few failures and was not able to identify any predictors [19]. Despite the fact that logistic regression was not feasible, we still could perform a linear regression analysis that identified that the number of preoperative reflux episodes on MII-pH monitoring is a significant determinant influencing the effect of LARS. Patients with a higher number of reflux episodes on MII-pH monitoring had significantly more reflux reduction after LARS. Age at the time of operation, neurodevelopment and type of fundoplication did not show a significant effect. In the adult literature, preoperative delayed GE negatively influenced the success of LARS [45, 46]. In children with GERD, delayed GE may influence the severity of GERD [47, 48]. Therefore, for this study we hypothesized that preoperative delayed GE could be a risk factor for failure of LARS in our pediatric cohort. In linear regression analysis, GE was, however, not a significant predictor. LARS did significantly improve GE in patients with preoperative delayed or severely delayed GE, which has also been demonstrated in adults [47, 48] and children [49] that have undergone LARS.

One of the limitations of this current study was that we enrolled fewer patients than anticipated. Although most results on the efficacy of LARS showed statistically significant differences, the number of included patients limited our linear regression analysis and therefore we were only able to investigate five determinants assuming enough statistical power with five included patients per chosen predictor. As only one patient failed after LARS, a logistic regression analysis to identify predictors of failure was not possible. Furthermore, 3-month follow-up time may be too short. As published in the previous study [9], reflux symptoms may increase over time, and therefore, it is important that we closely follow-up this current group over the years.

In conclusion, LARS significantly reduces reflux complaints, total AET and number of (acidic) reflux episodes in children with therapy-resistant GERD. LES resting pressure increases significantly after LARS, but esophageal function was not affected by the procedure. GE significantly improved in patients with preoperative delayed gastric emptying, but in the overall group no differences were observed. LARS showed better reflux reduction in children with a higher number of reflux episodes on preoperative MII-pH monitoring. Identifying predictors for failure was not possible due to the low failure rate of LARS in this cohort at 3-month follow-up. Future studies should entail multicenter prospective trials with a higher number of patients and long-term follow-up to assess parameters in predicting success of therapy.
